# Polyvinylpyrrolidone as a Stabilizer in Synthesis of AgInS_2_ Quantum Dots

**DOI:** 10.3390/nano12142357

**Published:** 2022-07-09

**Authors:** Oleg Korepanov, Olga Aleksandrova, Dmitrii Firsov, Zamir Kalazhokov, Demid Kirilenko, Dmitriy Kozodaev, Vasilii Matveev, Dmitriy Mazing, Vyacheslav Moshnikov

**Affiliations:** 1 Micro- and Nanoelectronics Department, Saint Petersburg Electrotechnical University “LETI”, 197022 Saint Petersburg, Russia; oaaleksandrova@gmail.com (O.A.); d.d.firsov@gmail.com (D.F.); kozodaev@ntmdt.nl (D.K.); dmazing@yandex.ru (D.M.); vamoshnikov@mail.ru (V.M.); 2 Institute of Physics and Mathematics, Kabardino-Balkarian State University, n.a. Kh.M. Berbekov, 360004 Nalchik, Russia; z-kalazh@yandex.ru; 3 Institute of Computer Science and Problems of Regional Management, Kabardino-Balkar Scientific Center, Russian Academy of Science, 360004 Nalchik, Russia; 4 Ioffe Institute, 194021 Saint Petersburg, Russia; zumsisai@gmail.com; 5 NT-MDT BV, 7335 Apeldoorn, The Netherlands; 6 Petersburg Nuclear Physics Institute Named by B.P.Konstantinov of NRC «Kurchatov Institute», 188300 Gatchina, Russia; matveev_va@pnpi.nrcki.ru

**Keywords:** quantum dots, AgInS_2_, polyvinylpyrrolidone, PVP

## Abstract

A synthesis protocol of polyvinylpyrrolidone-capped AgInS_2_ quantum dots in aqueous solution is reported. Nanoparticle morphology and chemical composition were studied by means of TEM, XRD, XPS, and FTIR. The obtained quantum dots were luminescent in the visible range. The photoluminescence intensity dependence on the polyvinylpyrrolidone amount was demonstrated. The wavelength of the emission maximum varied with changing the [Ag]:[In] molar ratio. The temperature dependence of the photoluminescence intensity of the polyvinylpyrrolidone-capped AgInS_2_ quantum dots was investigated within the temperature range of 11–294 K.

## 1. Introduction

Colloidal quantum dots (QDs) are semiconductor nanocrystals that undergo quantum confinement in three spatial dimensions. This results in the discretization of the energy levels and makes it possible to vary the photoluminescence (PL) and absorption energy with the changing nanocrystal size [1]. QDs are the object of intensive research in nanotechnology areas due to their potential applicability in solar energy, optoelectronics, sensorics, biomedicine, etc. [2,3,4,5,6]. Nanocrystals of binary semiconductors, e.g., II−VI (CdSe, PbSe, PbTe) and III−V (InP, InAs, GaAs, GaP), are well-studied materials. However, the toxic elements such as cadmium, lead, and arsenic in these compounds severely limit their widespread use, especially in areas related to biomedical applications. The search for safe, alternative materials for QDs led to the development of cadmium and lead-free I–III–VI compounds. Ternary metal chalcogenides such as CuInS_2_ and AgInS_2_ and the corresponding selenide analogues are promising materials for the wide range of implementations in optoelectronic devices and biosensing systems [2,3,4,5,6].

A promising trend in the technology of colloidal QDs is the development of methods in aqueous solutions, where a prerequisite is the use of hydrophilic ligands: L-glutathione, mercaptopropionic acid, mercaptoacetic acid, etc. [7]. This article presents a technique for synthesizing AgInS_2_ QDs stabilized with polyvinylpyrrolidone (PVP), a non-toxic, water-soluble linear polymer composed of 1-vinyl-2-pyrrolidone monomers [8]. PVP finds application in cosmetics, food, textiles, pharmaceuticals such as binders and coatings, solubilizers for suspensions, and solutions for disinfectant adsorbent [9]. PVP capping of I–III–VI QDs could expand the application of these QDs in biomedicines such as biosensing and bioimaging.

To the best of our knowledge, there are no published reports on the direct synthesis of I–III–VI QDs using PVP as the capping ligand. PVP has been used as a stabilizer of CdS and CdSe QDs [10,11]. In one work [12], the layers for optoelectronic devices were formed using PVP with L-glutathione-capped I–III–VI QDs being dispersed in the polymer matrix.

## 2. Materials and Methods

### 2.1. Chemicals

Indium(III) chloride (InCl_3_, 99.9%) was purchased from Sigma Aldrich (St. Louis, MO, USA). Silver nitrate (AgNO_3_, 99.5%), sodium sulfide (Na_2_S·9H_2_O, 98%), and Poly(N-vinyl-2-pyrrolidone) (av. M_w_ 12,600) were provided by Vekton (St. Petersburg, Russia). All chemicals were used without additional purification.

### 2.2. Synthesis of PVP-Capped AgInS_2_ QDs

PVP-capped AgInS_2_ QDs were synthesized in an aqueous solution. Under magnetic stirring, 0.01 mmol (1.7 mg) of AgNO_3_ and 8 mg of PVP were dissolved in 5 mL of deionized water with the subsequent addition of 0.04 mmol (8.8 mg) of InCl_3_ and heating up to 95 °C. The nucleation was performed by the rapid injection of 0.2 mmol (48 mg) of Na_2_S·9H_2_O; after that, the solution was heated for 1 h. The obtained suspensions were cooled to room temperature and subjected to centrifugation to remove large precipitates.

The molar ratio [Ag]:[In] was 1:4. The concentration ratio of PVP and sulfur anions to silver was set as equal to the varied molar ratio [Ag]:[In] (1:1, 1:2, 1:4, and 1:6).

### 2.3. Methods

Transmission electron microscopy (TEM) studies were performed using a Jeol JEM-2100F microscope (accelerating voltage 200 kV, point resolution 0.19 nm). Specimens for the TEM were prepared by the wetting of a conventional copper TEM grid covered with a carbon lacey film in the suspension and subsequently drying it in air.

The crystal structure of colloidal AgInS_2_ QDs was studied by X-ray diffraction (XRD). The diffraction patterns were obtained with a Rigaku SmartLab diffractometer (CuKα, 45 kW, 200 mA). The measurements were made in Bragg–Brentano geometry (θ/2θ) with a quasi-parallel X-ray beam formed by a Goebel mirror and parallel-slit collimator. To reduce the distortion of the diffraction peak profiles, a Soller slit (0.228 deg) was installed in front of the detector.

X-ray photoelectron spectroscopy (XPS) spectra were recorded on a “K-Alpha” Thermo Scientific system equipped with an Al-Kα (1486.6 eV) X-ray source. The sample was outgassed to less than 4.5 × 10^−9^ mbar. The background subtraction of secondary electrons was performed using the Shirley method. The spectrometer energy scale was calibrated with Au 4f_7/2_, Cu 2p_3/2_, Ag 3d_5/2_, with lines at 84.2, 367.9, and 932.4 eV, respectively.

The infrared transmission measurements were performed using a Vertex 80 Fourier-transform infrared (FTIR) spectrometer equipped with an SiC globar as the IR radiation source, a KBr beamsplitter, and a pyroelectric deuterated L-alanine-doped triglycine sulphate (DLaTGS) photodetector. A PL measurement setup [13] based on the same FTIR spectrometer (equipped instead with a CaF_2_ beamsplitter and a Si diode photodetector) was employed to obtain the temperature dependences of the PL spectra. The samples were placed into a Janis CCS-150 closed-cycle helium cryostat, and a 405-nm semiconductor laser diode was used as an excitation source. Nanoparticle samples were dried, powdered, and made into transparent films by mixing them with KBr.

PL spectra measurements of the QDs’ aqueous solutions were carried out on a spectrofluorometer with an MDR-206 monochromator using a silicon diode and a 405 nm semiconductor laser with a power of 10 mW at room temperature. Absorption spectra were acquired using a PE-5400UV UV-vis spectrophotometer (LLC “Ekohim”).

## 3. Results and Discussion

### 3.1. Structure Characterization

**Figure 1:**
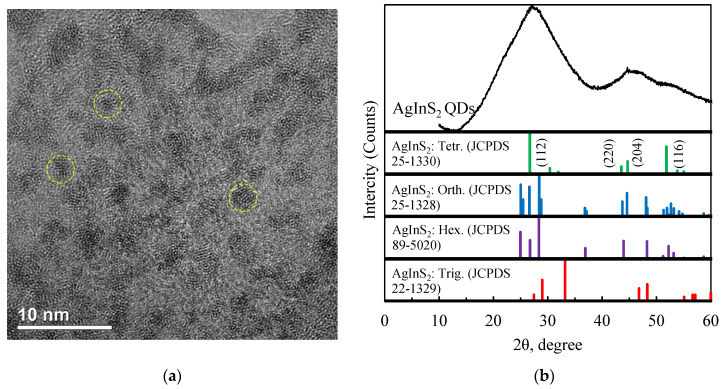
(**a**) TEM image of colloidal AgInS_2_ QDs; (**b**) XRD patterns of AgInS_2_ QDs.

The crystallinity of AgInS_2_ QDs was confirmed by TEM and XRD (Figure 1). The broad diffraction peaks were caused from the small size of the nanoparticles.

In the TEM image (Figure 1a), it can be observed that the particles of AgInS_2_ are approximately spherical. The average particle diameter is about 2 nm, which is smaller than the radius of the Bohr exciton in AgInS_2_. Thus, the nanoparticles should manifest quantum confinement effects.

The XRD pattern of AgInS_2_ QDs (Figure 1b) exhibited three broad peaks at 2θ values of 28.5, 46.5, and 52.5°. The average crystallite size (D) of the sample was calculated using Scherrer’s formula, which can be given as:

D = (K ∙ λ)/(β ∙ cos(θ)) (1)

where K is the shape factor (0.90), λ is the wavelength of the Cu Kα radiation (λ = 1.5406 Å), β is the full width at half maximum, and θ is the diffraction angle.

The average QD’s size of 1–2 nm estimated by XRD is in an agreement with the TEM results. The broad peaks observed indicate that the nanoparticles are short-range order nanostructures. AgInS_2_ QDs prepared at low temperatures are more likely to exist in the tetragonal than in the orthorhombic phase [14]. The agreement of the observed Bragg peaks with peaks corresponding to (112), (220), (204), and (116) planes of tetragonal AgInS_2_ (JCPDS 25-1330) indicates that the resulting particles were nuclei of a nascent tetragonal crystalline phase.

The exact formation mechanism of nanocrystals in the presence of PVP was not clear. In the work on the synthesis of noble metal nanocrystals stabilized by PVP, the coordination between Ag^+^ and PVP via an amide group was suggested, and the researchers distinguished two stages: the reduction in metal ions to neutral atoms, leading to the formation of clusters, and the coordination of the polymer to the metal cluster [8].

**Figure 2:**
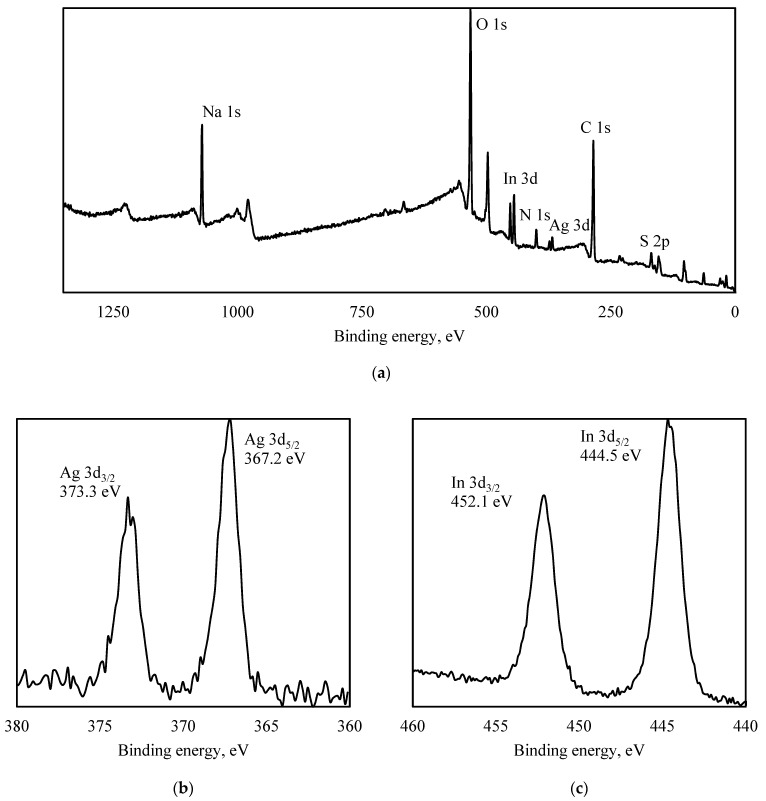

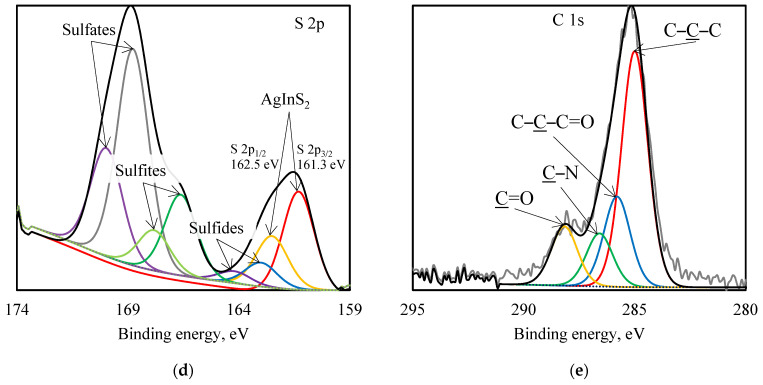
XPS spectra of PVP-capped AgInS_2_ QDs: survey (**a**), Ag 3d (**b**), In 3d (**c**), S 2p (**d**), C 1s (**e**).

In Figure 2, XPS spectra of PVP-capped AgInS_2_ QDs are shown.

From Figure 2a, it can be seen that Ag 3d peaks were found at 367.2 eV (Ag 3d_5/2_) and 373.3 eV (Ag 3d_3/2_), In 3d peaks were found at 444.5 eV (In 3d_5/2_) and 452.1 eV (In 3d_3/2_), and S 2p peaks were found at 162.5 eV (S 2p_1/2_) and 161.3 eV (S 2p_3/2_). As shown in the survey spectrum, the main peaks belonged to Ag, In, S, C, N, Na, and O elements.

The sulfur atoms formed a complex chemical composition. The peak decomposition is shown in Figure 2d. The S 2p_3/2_ peak at 161.2 eV showed that about 24 at.% is a part of the three-component AgInS_2_ system [15]. The S 2p peak can also be described by considering other chemical states involving small amounts of metal sulfides forming the film (162.4 eV, indium sulfide [16]) as well as significant amounts of compounds with sodium (166.4 eV, disodium sulfite [17], and 168.7 eV, sodium sulphate [18]).

Nitrogen has one definite chemical state with a binding energy of 399.7 eV, which corresponds to N 1s binding energy in PVP [19,20]. The lack of splitting for the N 1s line did not allow us to make a conclusion about the nitrogen involvement in the surface coordination.

The deconvolution of the C 1s line is analogous to [21]. The peaks with binding energies of 285.1, 285.8, 286.6, and 288.2 eV correspond to carbon in the C–C–C, C–C–C=O, C–N, and C=O states in PVP [21]. A significant difference in intensity was found only for the peak describing the C-N bond in this experiment.

**Figure 3:**
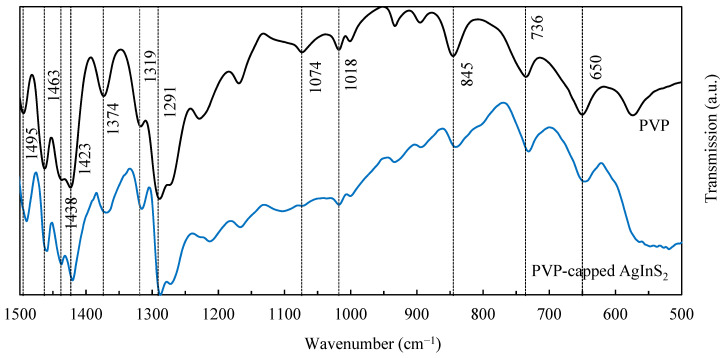
FTIR spectra of PVP and PVP-capped AgInS_2_ QDs.

In Figure 3, FTIR spectra of PVP and PVP-capped AgInS_2_ QDs are presented.

The absorption bands that appeared at 1291, 1319, 1374, 1423, 1438, 1463, and 1495 cm^−1^ were assigned to the vibration in the PVP heterocycle [22]. The bands at 1074 [23] and 1018 cm^−1^ were the result of the C–N stretching vibration; the bands at 845, 736, and 650 cm^−1^ provided information about the out-of-plane C–H bending, rocking, and OH wagging, respectively.

It is not entirely clear which coordination mechanism is responsible for nanoparticles stabilization. In [24], the authors concluded that both nitrogen and oxygen of the pyrrolidone ring can be coordinated to the silver and gold surfaces. Analyzing the pyrrolidone ring structure, the authors proposed that two potentially chemisorbing sites are available: a carbonyl oxygen and the nitrogen. They proposed two models of the interaction process between the pyrrolidone molecule and the silver or gold nanoparticle surface: a pyrrolidone molecule chemisorbing to a silver and gold atom through the carbonyl oxygen and a pyrrolidone molecule chemisorbing through both carbonyl oxygen and nitrogen atoms, although the latter mechanism is constrained by steric factors [24]. In [20], where PVP-capped palladium nanoparticles were studied, the authors came to the conclusion that nitrogen coordination sites compared to oxygen ones become more significant with the increase in the nanoparticle size, indicating a preferential oxygen participation in stabilization. In the case of ternary I–III–VI systems, PVP coordination is likely to be more complex. The results of XPS and FTIR spectroscopy did not allow us to determine the coordination mechanism in PVP-capped AgInS_2_ QDs in this work.

### 3.2. Absorption and PL Spectra

**Figure 4:**
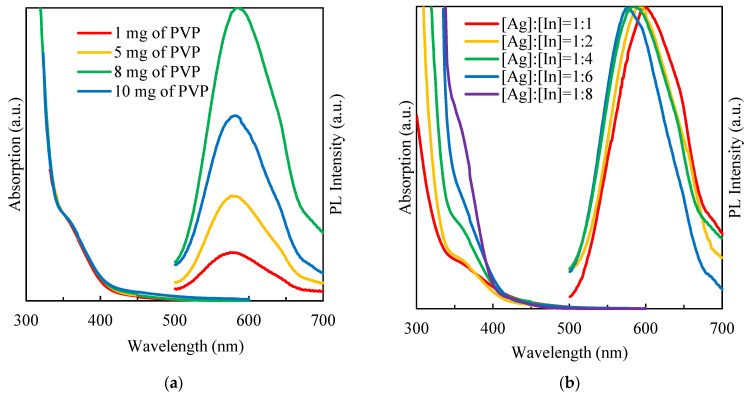
Absorption and PL spectra of AgInS_2_ QDs solutions prepared with different (**a**) PVP concentrations, (**b**) molar ratio of [Ag]:[In].

The amount of surfactant was varied for QDs’ synthesis with the molar ratio of [Ag]:[In] = [1]:[4]. The synthesized colloidal AgInS_2_ QDs were luminescent with a PL maximum at ~585 nm and with a full width at half maximum (FWHM) of ~110 nm (Figure 4).

Varying the amount of PVP in solution did not lead to a significant shift of the PL peak position but affected the emission intensity. According to the obtained results, the strongest PL was observed for QDs synthesized with an amount of PVP equal to 8 mg per 0.004 mmol [In^3+^]. As the PVP concentration in the solution increased, the passivation efficiency of the QDs’ surface was likely to increase, leading to a reduction in non-radiative emission centers [10]. The same trend of reducing the PL intensity was observed in the synthesis of L-glutathione-capped AgInS_2_ QDs [25]. The authors supposed that an excessive ligand concentration might result in the surface distortion, which originates nonradiative defects, reducing PL intensity, while there was no change in PL peak position [25].

The absorption properties were assessed by UV-vis spectroscopy. Figure 4a shows the absorption spectra of AgInS_2_ QDs at different amounts of PVP. A broad and featureless absorption curve with a long absorption tail in the low-energy area without clearly defined excitonic maxima were observed for all samples [26,27]. The variation of the PVP concentration did not affect either the form or the position of the absorption band.

The PL measurement results presented in Figure 4b are in good agreement with similar works where these types of QDs were stabilized using L-glutathione [28] or mercaptopropionic acid [29]. The variation of the molar ratio between metals of groups I and III in I–III–VI QDs led to a change in the energy gap and the PL peak position [26,27]. The decrease in the molar ratio [Ag]:[In] may lead to a change of the predominant defect-related luminescent channel as well as to a widening of the band gap. A systematic blue shift of emission observed in the QDs’ samples with a higher degree of Ag deficiency should be associated with these two factors. As seen from the normalized PL spectra of AgInS_2_ QDs (Figure 4b), all samples were emitted in the yellow region (with peak wavelengths ranging from 597 to 575 nm for an [Ag]:[In] ratio changing from 1:1 to 1:6 with a broad FWHM of 100–120 nm).

**Figure 5:**
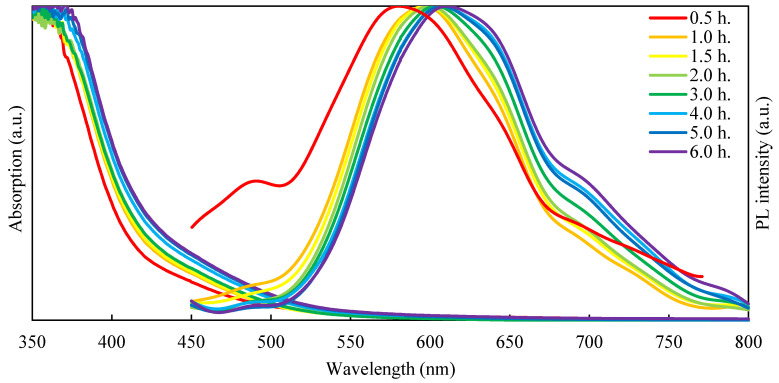
PL and absorption spectra of AgInS_2_ QDs obtained at different intervals after nucleation.

The time-dependent synthesis was carried out with a slight protocol modification: the QDs were synthesized in a three times higher volume and three times higher concentration. At certain time intervals after nucleation, 1 mL aliquots were withdrawn from the solution for PL and absorption properties’ characterization. Figure 5 shows the PL and absorption spectra of AgInS_2_ QDs with a molar ratio of [Ag]:[In] = [1]:[4] obtained at different times during the synthesis.

As the synthesis time increase, nanoparticles grow in size, leading to a reduction in quantum confinement, which is expressed as red shift in both absorption and PL spectra [30].

The mechanism of PL in the I–III–VI QDs is a subject of ongoing debate. Different researchers have ascribed it to the processes of radiative recombination using the donor–acceptor pair (DAP) model [31], exciton fine-structure model [32], or recombination of a localized hole and a delocalized electron [33]. Finite depth well effective mass approximation calculations show that photoexcited electrons occupy an intraband donor state before recombination, which confirms the validity of the DAP theory [34]. In AgInS_2,_ QDs’ sulfur vacancies (V_S_) and silver antisites (In_Ag_) as donor levels and silver vacancies (V_Ag_) as acceptor levels are distinguished among the probable defects [35]. The effective emission through the intraband states in ternary QDs makes them different from binary ones in terms of optical properties. One of the specific features of the I–III–VI QDs is the possibility of adjusting the optical properties through cation molar ratio variation.

### 3.3. Temperature-Dependent Photoluminescence

The temperature-dependent PL measurement was performed between 11 and 294 K under the 405 nm laser excitation. The PL spectrum of AgInS_2_ QDs in a dielectric matrix has a more complicated shape compared to the previously obtained PL spectrum of AgInS_2_ QDs in solution. Two regions can be distinguished: the first with an intensive peak at ~670 nm and the second with a peak at ~900 nm. The dielectric matrix may provide additional defect states at the interface, which could have different radiant energies than the exciton recombination in QDs [36]. The intensity of a low-energy PL band compared to a high-energy PL band decreases more rapidly with the rising temperature, which causes a slight red shift of the entire PL curve. In [37], the authors presented the temperature-dependent PL measurements’ results for CdTe QDs of different sizes. For the smallest ones (2.3 nm), the appearance of the low-energy band took place at higher temperatures, which was not observed at all for larger particles (3 nm) in the temperature range of 360–120 K. This finding confirmed the influence of the surface states on the PL properties (the smaller the particle size, the higher the ratio between volume and surface area). There was practically no change in the PL peak position and FWHM of the high-energy band.

**Figure 6:**
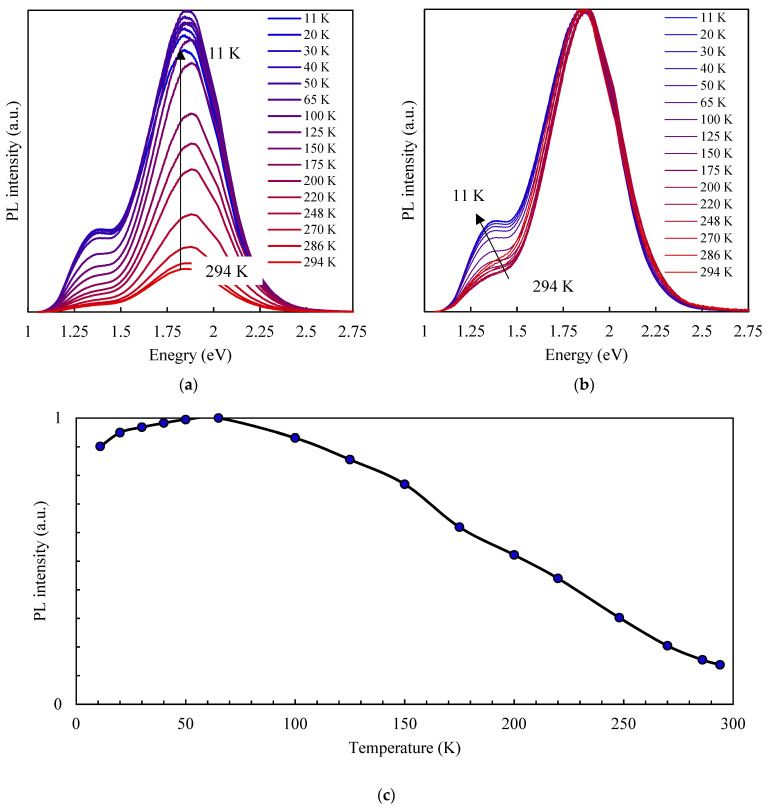
Temperature-dependent PL spectra (**a**), normalized temperature-dependent PL spectra (**b**), and temperature dependence of the PL integral intensity (**c**) of PVP-capped AgInS_2_ QDs.

Increasing the temperature from 11 to 65 K resulted in PL enhancement, and a further temperature increase led to linear PL quenching (Figure 6c). A similar dependency was observed by Lifshitz et al. [38] for a trap-related emission band in CdSe nanoparticles. The authors suggested that the PL intensity growth up to 50 K observed in their samples can be explained by a changing population of deep traps accompanied by carriers de-trapping from exciton states and followed by carriers re-trapping in deeper states with an increasing temperature. Hamanaka et al. [35] similarly observed an increase in the PL intensity in the low-temperature range in AgInS_2_ QDs. They applied their model and explained further PL quenching at higher temperatures by thermal depletion of donor and acceptor levels and an enhancement of nonradiative recombination of photoexcited carriers. Another possible explanation for PL intensity enhancement with rising temperature in the low-temperature range is the increasing charge carriers’ mobility, which provides thermal activation of the recombination pathways [38].

## 4. Conclusions

PVP-capped AgInS_2_ QDs were successfully synthesized in an aqueous solution. The average particle size was about 2 nm. All obtained samples were luminescent in the visible region with a broad FWHM of 100–120 nm. Varying the amount of PVP in the solution did not significantly shift the PL peak position or change the absorption curve but did affect the emission intensity. The maximum PL intensity was reached at a PVP concentration of 8 mg per 0.04 mmol of indium cations. By controlling the synthesis time, it was feasible to modify the optical properties of the forming particles: with increasing synthesis time, the PL peak position shifted to lower energies. By adjusting the [Ag]:[In] molar ratio in AgInS_2_ QDs, the PL peak position can be tuned from 597 to 575 nm. A temperature increase from 11 to 65 K resulted in PL enhancement, and further temperature increases resulted in linear PL quenching.
